# Time to Clinical Benefit of Intensive Blood Pressure Lowering in Patients 60 Years and Older With Hypertension: A Secondary Analysis of Randomized Clinical Trials

**DOI:** 10.1001/jamainternmed.2022.1657

**Published:** 2022-05-09

**Authors:** Tao Chen, Fang Shao, Kangyu Chen, Yang Wang, Zhenqiang Wu, Yongjuan Wang, Yanpei Gao, Victoria Cornelius, Chao Li, Zhixin Jiang

**Affiliations:** 1 Department of Public Health, Policy and Systems, Institute of Population Health, The University of Liverpool, Liverpool, United Kingdom; 2 Department of Clinical Sciences, Liverpool School of Tropical Medicine, Liverpool, United Kingdom; 3 Department of Biostatistics, School of Public Health, Nanjing Medical University, Jiangsu, Nanjing, China; 4 Department of Cardiology, The First Affiliated Hospital of USTC, Hefei, China; 5 Division of Life Sciences and Medicine, University of Science and Technology of China, Hefei, China; 6 Medical Research and Biometrics Center, Fuwai Hospital, National Center for Cardiovascular Disease, Peking Union Medical College and Chinese Academy of Medical Sciences, Mentougou District, Beijing, China; 7 Department of Geriatric Medicine, The University of Auckland, Auckland, New Zealand; 8 Department of Epidemiology and Health Statistics, School of Public Health, Xi’an Jiaotong University Health Science Center, Xi’an, China; 9 Imperial Clinical Trials Unit, School of Public Health, Imperial College London, London, United Kingdom; 10 Department of Cardiology, The First Affiliated Hospital of Nanjing Medical University, Jiangsu Province Hospital, China

## Abstract

**Importance:**

Recent guidelines recommend a systolic blood pressure (BP) goal of less than 150 mm Hg or even 130 mm Hg for adults aged 60 years or older. However, harms from intensive BP treatments occur immediately (eg, syncope, fall), and benefits for cardiovascular event reduction emerge over time. Therefore, harms with low chance of benefit need to be clearer, particularly for those with limited life expectancy.

**Objective:**

To estimate the time needed to potentially derive clinical benefit from intensive BP treatment in patients 60 years and older.

**Design, Setting, and Participants:**

This secondary analysis included individual patient data from published randomized clinical trials with 27 414 patients 60 years or older with hypertension. Patient-level survival data were reconstructed when the original data were not available. Published trials were identified by searching PubMed until October 15, 2021.

**Exposures:**

Intensive BP lowering vs standard BP lowering with the treat-to-target design.

**Main Outcomes and Measures:**

Major adverse cardiovascular event (MACE) defined by each trial, which was broadly similar with all trials including myocardial infarction, stroke, and cardiovascular mortality.

**Results:**

Six trials (original data from 2 trials and reconstructed data from 4 trials) with 27 414 participants (mean age, 70 years; 56.3% were women) were included in the analysis. Intensive BP treatment with a systolic BP target below 140 mm Hg was significantly associated with a 21% reduction in MACE (hazard ratio, 0.79; 95% CI, 0.71-0.88; *P* < .001). On average, 9.1 (95% CI, 4.0-20.6) months were needed to prevent 1 MACE per 500 patients with the intensive BP treatment (absolute risk reduction [ARR], 0.002). Likewise, 19.1 (95% CI, 10.9-34.2) and 34.4 (95% CI, 22.7-59.8) months were estimated to avoid 1 MACE per 200 (ARR, 0.005) and 100 (ARR, 0.01) patients, respectively.

**Conclusions and Relevance:**

In this analysis, findings suggest that for patients 60 years and older with hypertension, intensive BP treatment may be appropriate for some adults with a life expectancy of greater than 3 years but may not be suitable for those with less than 1 year.

## Key Points

**Question:**

What is the time to benefit of intensive blood pressure treatment in adults 60 years and older?

**Findings:**

In this analysis of 6 randomized clinical trials consisting of 27 414 individuals with hypertension aged 60 years and older, 19.1 months and 34.4 months were needed to avoid 1 cardiovascular event for 200 and 100 patients, respectively.

**Meaning:**

These findings suggest that intensive blood pressure treatment may be appropriate for some adults with hypertension aged 60 years and older with a life expectancy of greater than 3 years but may not be suitable for those with less than 1 year.

## Teaser

This secondary analysis of randomized clinical trials estimates the time needed to potentially derive clinical benefit from intensive blood pressure treatment in patients 60 years and older.

## Introduction

Although there are conflicting guideline recommendations for the control of blood pressure (BP), evidence from a meta-analysis of 4 trials in patients with hypertension 65 years and older has shown that intensive BP control could reduce the risk of cardiovascular events.^1^ This was supported by another 2 meta-analyses demonstrating that a lower systolic BP was associated with better cardiovascular outcomes across all age groups.^2,3^ More recently, results of patients aged 60 to 80 years from the STEP (Strategy of Blood Pressure Intervention in the Elderly Hypertensive Patients) trial indicated that intensive treatment to target a systolic BP of 110 to 130 mm Hg reduced the risk of cardiovascular events compared with standard treatment with a target of 130 to 150 mm Hg.^4^

Patients aged 60 years and older usually have tremendous heterogeneity in cardiovascular risk, such as diabetes, poor kidney function, left ventricular hypertrophy, and hyperlipidemia. Thus, clinicians need to individually weigh benefits against potential risks (eg, acute kidney injury, hypotension, syncope, falls, electrolyte abnormalities) when considering intensive BP control.^5,6^ Because harms of treatments can occur immediately, but benefits emerge over time, a framework for individualizing prevention treatment decisions incorporating this lag time to benefit (TTB) has been discussed, and it has been argued that patients with limited life expectancy were exposed to the potential harms of prevention with little chance of benefit.^7,8^ Limited studies have estimated the TTB for statins,^9^ breast and colorectal cancer screening,^10,11^ and bisphosphonate therapy.^12^ It remained unclear how long a patient needed to live to potentially benefit from intensive BP control in patients 60 years and older. As such, to help clinicians individualize BP control therapy among older patients with hypertension, we conducted an analysis of individual participant data on the basis of the available evidence from randomized clinical trials to determine the TTB of more vs less intensive BP control.

## Methods

### Institutional Review Board and Patient Consent

The Xi’an Jiaotong University Health Science Center institutional review board approved this study. Patient consent was not required for this secondary data analysis.

### Data Source and Searches

We performed this study on the basis of up-to-date published research. This study followed the Preferred Reporting Items for Systematic Reviews and Meta-analyses (PRISMA) reporting guidelines. Two independent reviewers (Yongjuan W. and Y.G.) searched previous systematic reviews and meta-analyses in PubMed to identify published clinical trials of intensive BP in the older adult patients with hypertension. We also searched subsequently published relevant trials until October 15, 2021. We adopted the search terms used in the systematic review performed by Bavishi et al,^1^ which included the following Medical Subject Headings (of the US National Library of Medicine) terms: *randomized controlled trial*, *target BP*, *goal BP*, *intensive BP*, *tight BP*, *elderly*, and *older patients*.

In our study, we only included randomized clinical trials that (1) compared intensive BP lowering vs standard BP lowering with treat-to-target designs (eg, systolic BP <120 mm Hg vs <140 mmHg), but not those with placebo as a control arm because these trials were structured to answer separate and largely incompatible clinical questions; (2) enrolled patients 60 years and older with hypertension because these patients may experience the up-front harms from the intensive BP treatment with little chance that they survive to receive the benefit; (3) provided follow-up data on cardiovascular events; and (4) had vector Kaplan-Meier (KM) curves to extract individual participant time-to-event data if original individual data could not be accessed.

### Data Reconstructing Process

Individual data were reconstructed from the numbers of patients at risk and the KM graph when original study data were not available. Basically, the reconstruction was a 2-stage process. Stage 1 was to extract quality data coordinates (survival probability and time) from KM curves by DigitizeIt software (https://www.digitizeit.xyz/) following the instructions from Liu et al^13^ and Guyot et al.^14^ After extracting the raw data of time and survival probability, a STATA function developed by Wei and Royston^15^ was used to rebuild the individual data (stage 2). This validated algorithm has been used in various research.^16,17^ We presented the side-by-side comparisons of reconstructed and original curves (see eFigures 1-6 in the Supplement) and visually found that the algorithm recovered individual participant data from published trials with a high degree of accuracy.

### Primary Outcomes

The primary outcome is time to the first major adverse cardiovascular event (MACE), originally defined by individual trial as a composite of cardiovascular outcomes. Although the definitions of MACE differed across the included trials, they were broadly similar because all trials included myocardial infarction, stroke, and cardiovascular death. Detailed components of MACE in each trial are shown in eTable 2 in the Supplement.

### Statistical Analysis

The cumulative MACE rates at each time point in the standard and intensive BP control groups from the included trials were estimated using stratified KM curves. To address the between-study heterogeneity arising due to the clustering of participants at the study-level, the hazard ratio (HR) and its 95% CI were calculated using the stratified Cox proportional hazards model. The above analysis was repeated for different target systolic BPs (ie, <140 mm Hg, <130 mm Hg, and <120 mm Hg).

To estimate the time to specific absolute risk reduction (ARR) thresholds (ie, 0.002, 0.005, and 0.01), we fitted Weibull survival curves using the individual participant data. We calculated TTB and its CI using the conventional frequentist method with Monte Carlo simulations. The details of these procedures are reported in eMethods in the Supplement. To consider the heterogeneity of the included studies, we presented TTB for the following categories: trials that did not include participants with diabetes only; trials that did not include kidney outcomes as MACE outcomes; trials that only included participants older than 60 years; trials where the intervention targeted systolic BP (<130 mm Hg or <140 mm Hg); and trials with targeted systolic BP less than 160 mm Hg as the usual care. The TTB calculation was conducted in R, version 3.4.0 (R Foundation for Statistical Computing), and other analyses in this study used Stata, version 15.0 (StataCorp LLC).

## Results

**Figure 1.**
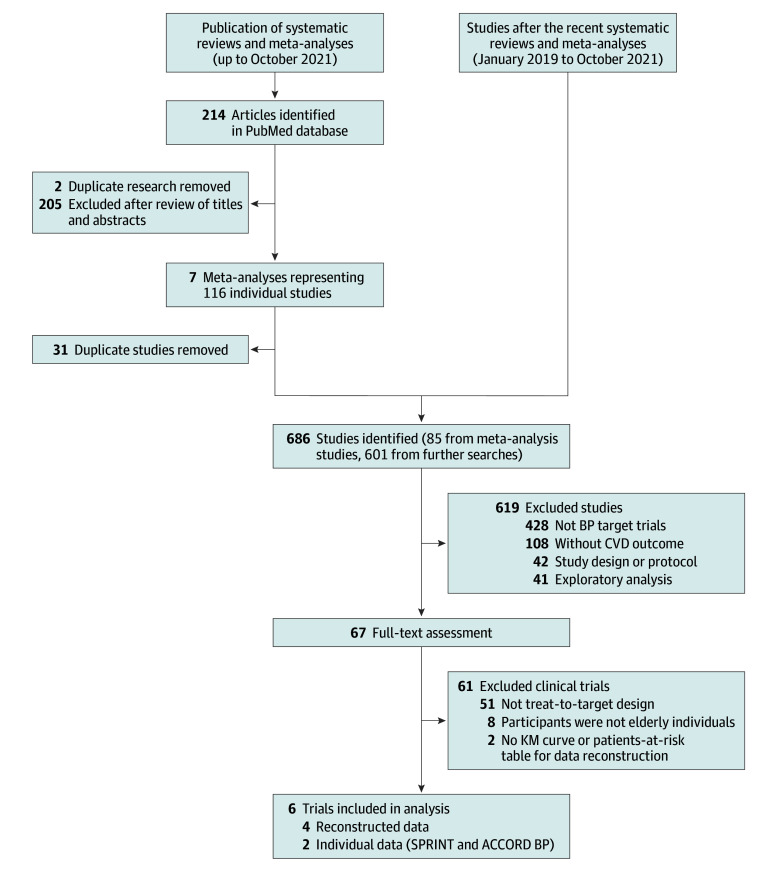
Flowchart of the Search, Selection, and Inclusion of the Studies ACCORD BP indicates Action to Control Cardiovascular Risk in Diabetes Blood Pressure trial; BP, blood pressure; CVD, cardiovascular disease; KM, Kaplan-Meier; SPRINT, Systolic Blood Pressure Intervention Trial.

We identified 85 trials from 7 systematic reviews and meta-analyses^1,18,19,20,21,22,23^ and an additional 601 studies after the latest previous electronic search included in the reviews. A total of 619 were excluded, and the remaining 67 underwent full-text assessment. Of them, 61 trials were excluded for the following reasons: not treat-to-target design (n = 51), participants were not older adults (n = 8), and no KM curves or patients-at-risk table for data reconstruction (n = 2). Finally, 6 trials were included in the analysis. Of them, original individual data from SPRINT (Systolic Blood Pressure Intervention Trial)^24^ and ACCORD BP (Action to Control Cardiovascular Risk in Diabetes Blood Pressure trial)^25^ were obtained through the National Heart, Lung, and Blood Institute on approval. We reconstructed the individual data for the remaining 4 trials: Cardio-Sis (Studio Italiano Sugli Effetti Cardiovascolari del Controllo della Pressione Arteriosa Sistolica),^26^ JATOS (Japanese Trial to Assess Optimal Systolic Blood Pressure in Elderly Hypertensive Patients),^27^ VALISH (Valsartan in Elderly Isolated Systolic Hypertension),^28^ and STEP.^4^ The design and results of the included clinical trials have been reported previously.^4,24,25,26,27,28^ The search results are illustrated in Figure 1, and the list of the excluded clinical trials with their exclusion reason is shown in eTable 1 in the Supplement.

The characteristics of the included trials are summarized in Table 1. Overall, there were 27 414 participants with 56.3% women. The mean age was 70 years, and the sample size ranged from 1111 to 8511. The intensive treatment was found to be significantly better than standard treatment in the SPRINT,^24^ Cardio-Sis,^26^ and STEP^4^ trials. Other characteristics of the included trials are shown in Table 1 and eTable 2 and eTable 3 in the Supplement. Their risk of bias assessment can be found in eTable 4 in the Supplement.

**Table 1.** Characteristics of Included Studies

Characteristic	Original data		Reconstructed data			
	SPRINT^24^^,^^a^	ACCORD BP^25^^,^^a^	Cardio-Sis^26^^,^^b^	JATOS^27^	VALISH^28^	STEP^4^
No. of participants	7398	2897	1111	4418	3079	8511
Published date	2015	2010	2009	2008	2010	2021
Study population	Patients without diabetes	Patients with diabetes	Patients without diabetes	Mixed patient population	Mixed patient population	Mixed patient population
Site performed	North America	North America	Italy	Japan	Japan	China
Age, mean (range), y	71.2 (60-90)	66.8 (60-79)	67 (>55)	73.6 (65-85)	76.1 (70-84)	66.25 (60-80)
Women, No. (%)	3332 (35.6)	2258 (47.7)	653 (58.8)	2701 (61.1)	1924 (62.5)	4560 (53.6)
Mean baseline SBP, mm Hg	139.8	140.4	163.3	171.6	169.6	146.1
BP treatment goal, mm Hg	Intensive: SBP <120 vs standard: SBP <140	Intensive: SBP <120 vs standard: SBP <140	Intensive: SBP *<*130 vs standard: SBP <140	Intensive: SBP <140 vs standard: SBP <160	Intensive: SBP <140 vs standard: SBP <150	Intensive: SBP <130 vs standard: SBP <150
Achieved SBP, mm Hg	121.4 vs 136.2	119.3 vs 133.5	131.9 vs 135.6	135.9 vs 145.6	136.6 vs 142.0	127.5 vs 135.3
Average/median follow-up, y	3.3	4.7	2.0	2.0	3.1	3.3
HR (95% CI) of MACE^c^	0.78 (0.65-0.93)^d^	0.89 (0.71-1.10)^d^	0.50 (0.13-0.79)	NA (0.77-1.42)^e^	0.89 (0.60-1.31)	0.74 (0.60-0.92)

Abbreviations: ACCORD BP, Action to Control Cardiovascular Risk in Diabetes Blood Pressure trial; BP, blood pressure; Cardio-Sis, Studio Italiano Sugli Effetti Cardiovascolari del Controllo della Pressione Arteriosa Sistolica; HR, hazard ratio; JATOS; Japanese Trial to Assess Optimal Systolic Blood Pressure in Elderly Hypertensive Patients; MACE, major adverse cardiovascular events; NA, not available; SBP, systolic BP; SPRINT, Systolic Blood Pressure Intervention Trial; STEP, Strategy of Blood Pressure Intervention in the Elderly Hypertensive Patients; VALISH, Valsartan in Elderly Isolated Systolic Hypertension.

^a^ Patients younger than 60 years from the SPRINT (n = 1963) and ACCORD BP (n = 1836) studies were excluded in the current analysis.

^b^ Few patients aged 55 to 60 years were included.

^c^ The MACE definition for each study was listed (see eTable 2 in the Supplement).

^d^ The HR was calculated using the Cox model from limited-access SPRINT and ACCORD BP BioLINCC data sets.

^e^ The original article included only the CI.

**Figure 2.**
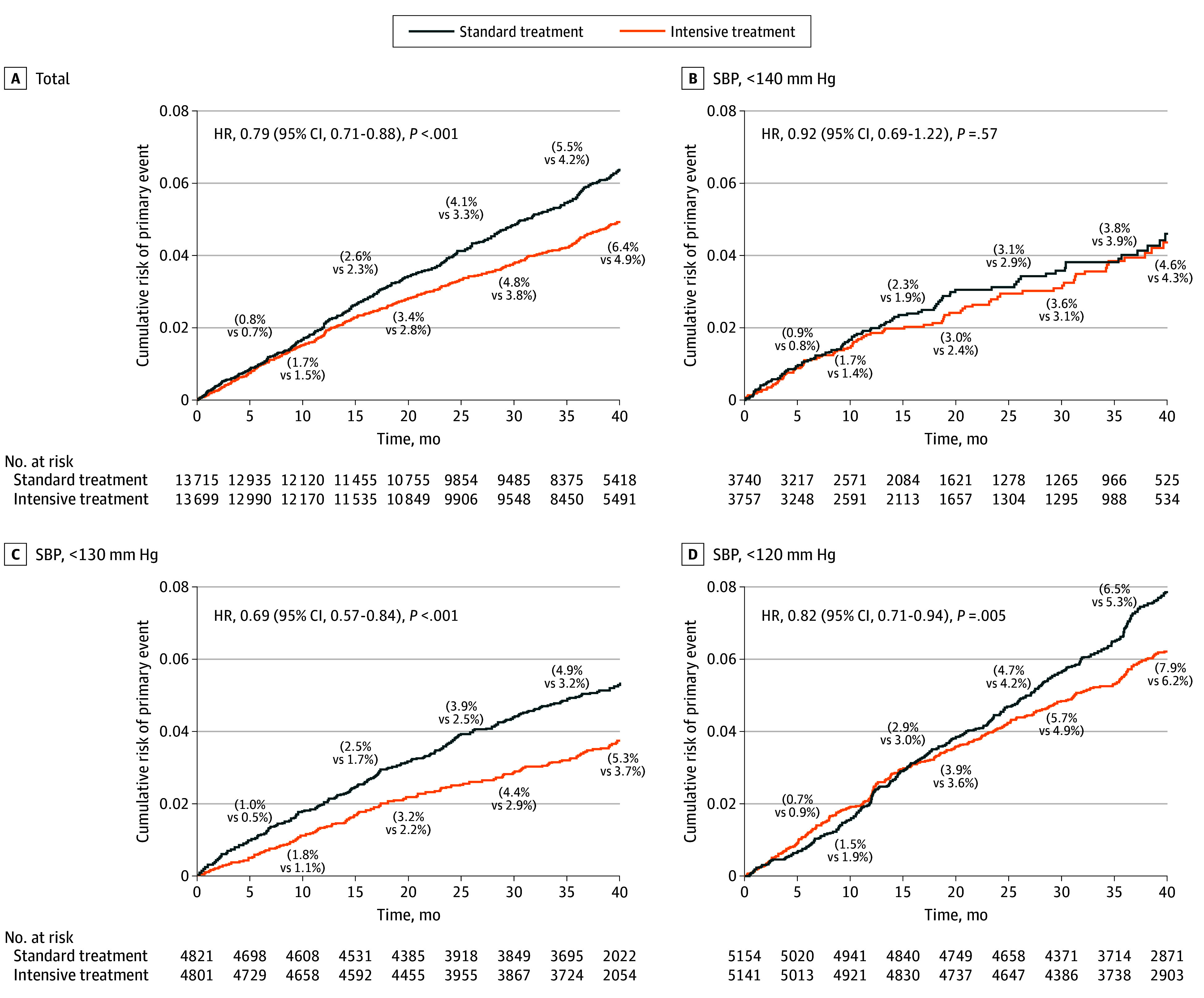
Cumulative Incidence of Major Adverse Cardiovascular Events in the Standard and Intensive Blood Pressure Treatment Groups Cumulative incidence and hazard ratios (HRs) (calculated by stratified Cox model) of primary event in the standard and intensive blood pressure treatment groups among total and different target systolic blood pressure (SBP) of intensive treatment strategy subgroups: total (A), target SBP less than 140 mm Hg (JATOS^27^ and VALISH^28^ studies) (B), target SBP less than 130 mm Hg (STEP^4^ and Cardio-Sis^26^ studies) (C), and target SBP less than 120 mm Hg (SPRINT^24^ and ACCORD BP^25^ studies) (D). ACCORD BP indicates Action to Control Cardiovascular Risk in Diabetes Blood Pressure trial; Cardio-Sis, Studio Italiano Sugli Effetti Cardiovascolari del Controllo della Pressione Arteriosa Sistolica; JATOS; Japanese Trial to Assess Optimal Systolic Blood Pressure in Elderly Hypertensive Patients; SPRINT, Systolic Blood Pressure Intervention Trial; STEP, Strategy of Blood Pressure Intervention in the Elderly Hypertensive Patients; VALISH, Valsartan in Elderly Isolated Systolic Hypertension.

The cumulative MACE rates at different time points can be found for all trials and by subgroups in Figure 2. The KM curves indicated a consistently lower cumulative incidence of MACE in intensive treatment vs the standard BP treatment overall (HR, 0.79; 95% CI, 0.71-0.88; *P* < .001) (Figure 2A). Although there was no statistically significant association for MACE between target SBP less than 140 mm Hg vs SBP less than 150/160 mm Hg (HR, 0.92; 95% CI, 0.69-1.22; *P* = .57) (Figure 2B), we found a significant treatment benefit in subgroups of target SBP less than 130 mm Hg vs SBP less than 150/140 mm Hg (HR, 0.69; 95% CI, 0.57-0.84; *P* < .001) (Figure 2C) and target SBP less than 120 mm Hg vs SBP less than 140 mm Hg (HR, 0.82; 95% CI, 0.71-0.94; *P* = .005) (Figure 2D).

Analyses to determine the TTB at different clinical meaningful thresholds indicated that 9.1 (95% CI, 4.0-20.6) months were needed to prevent 1 MACE per 500 patients with the intensive BP treatment (ARR = 0.002). Similarly, 19.1 (95% CI, 10.9-34.2) and 34.4 (95% CI, 22.7-59.8) months were estimated to avoid 1 MACE per 200 (ARR = 0.005) and 100 (ARR = 0.01) patients (Table 2). The TTB for individual trials is shown in eTable 5 in the Supplement. The TTB to specific ARR thresholds varied across different subgroups but with little changes compared with the overall estimate. However, the TTB was consistently higher in the subgroup of target SBP less than 120 mm Hg vs SBP less than 140 mm Hg compared with that of target SBP less than 130 mm Hg vs SBP less than 150/160 mm Hg (Table 3).

**Table 2.** Time to Benefit (Months) at Specific Thresholds of Absolute Risk Reduction^a^

Threshold	SPRINT^24^	+ACCORD BP^25^	+Cardio-Sis^26^	+JATOS^27^	+VALISH^28^	+STEP^4^
0.002	17.1 (1.1-19.5)	17.9 (1.9-29.9)	11.5 (2.5-23.6)	12.6 (3.1-26.4)	12.3 (4.0-28.0)	9.1 (4.0-20.6)
0.005	23.0 (5.3-38.7)	26.3 (11.2-49.4)	19.1 (8.8-35.9)	20.7 (10.3-41.2)	21.7 (11.3-43.1)	19.1 (10.9-34.2)
0.01	31.3 (16.6-55.4)	37.7 (22.6-69.7)	29.6 (17.6-49.9)	32.2 (19.7-60.4)	35.3 (21.7-67.8)	34.4 (22.7-59.8)

Abbreviations: ACCORD BP, Action to Control Cardiovascular Risk in Diabetes Blood Pressure trial; Cardio-Sis, Studio Italiano Sugli Effetti Cardiovascolari del Controllo della Pressione Arteriosa Sistolica; JATOS; Japanese Trial to Assess Optimal Systolic Blood Pressure in Elderly Hypertensive Patients; SPRINT, Systolic Blood Pressure Intervention Trial; STEP, Strategy of Blood Pressure Intervention in the Elderly Hypertensive Patients; VALISH, Valsartan in Elderly Isolated Systolic Hypertension.

^a^ Each study is added in succession starting from left to right, and the time to benefit is re-estimated with the far-right column being the summary time to benefit after including all studies.

**Table 3.** Time to Benefit (Months) in the Subgroups for the Different Thresholds

Included studies						Study characteristics	Time to benefit (95% CI)		
SPRINT^24^	ACCORD BP^25^	Cardio-Sis^26^	JATOS^27^	VALISH^28^	STEP^4^	Subgroup	0.002	0.005	0.01
						**Exclude:**			
Yes	No	Yes	Yes	Yes	Yes	Diabetes only	8.8 (3.7-19.3)	17.7 (10.1-31.3)	31.2 (20.9-54.0)
Yes	Yes	No	Yes	Yes	Yes	Cardio-Sis^a^	11.1 (4.6-25.7)	22.2 (12.5-41.8)	38.7 (25.3-71.6)
Yes	Yes	Yes	No	No	Yes	Target SBP <140 mm Hg	8.2 (3.4-19.6)	17.1 (9.3-30.6)	30.4 (19.7-49.3)
Yes	Yes	Yes	No	Yes	Yes	JATOS^b^	8.2 (3.6-19.4)	17.8 (10.1-32.1)	32.6 (21.4-53.6)
Yes	Yes	No	No	No	Yes	Kidney outcome	10.6 (4.1-24.8)	20.6 (11.1-37.5)	35.3 (22.7-58.7)
						**Include:**			
No	No	Yes	No	No	Yes	Target SBP <130 mm Hg	2.2 (0.9-9.2)	7.6 (3.3-23.5)	20.4 (9.8-188.6)
Yes	Yes	No	No	No	No	Target SBP <120 mm Hg	18.0 (1.9-30.0)	26.3 (11.2-49.4)	37.7 (22.6-69.7)
Yes	Yes	Yes	Yes	Yes	Yes	All trials	9.1 (4.0-20.6)	19.1 (10.9-34.2)	34.4 (22.7-59.8)

Abbreviations: ACCORD BP, Action to Control Cardiovascular Risk in Diabetes Blood Pressure trial; Cardio-Sis, Studio Italiano Sugli Effetti Cardiovascolari del Controllo della Pressione Arteriosa Sistolica; JATOS; Japanese Trial to Assess Optimal Systolic Blood Pressure in Elderly Hypertensive Patients; SBP, systolic blood pressure; SPRINT, Systolic Blood Pressure Intervention Trial; STEP, Strategy of Blood Pressure Intervention in the Elderly Hypertensive Patients; VALISH, Valsartan in Elderly Isolated Systolic Hypertension.

^a^ Few patients aged 55 to 60 years were included.

^b^ Usual care group with SBP target of less than 160 mm Hg.

## Discussion

To our knowledge, this is the first study to use robust quantitative methods to determine the TTB for the prevention of cardiovascular events with intensive BP control in patients 60 years and older. It fills a critical gap for individually weighing benefits against potential harms while considering intensive BP control in this population, especially those with limited life expectancy. In this study, we found strong evidence for intensive BP treatment (SBP goal of <140 mm Hg) to lower MACE events, and intensive BP treatment took 9, 19, and 34 months on average to prevent 1 MACE in 500, 200, and 100 patients, respectively, which suggested that intensive BP may be appropriate for older patients with a life expectancy greater than corresponding years after considering their adverse events (such as hypotension or falls) from more aggressive BP treatment.

The concept of intensive BP control has been extensively discussed. However, controversies still exist among the current guidelines for treating older patients. A BP target of less than 130/80 mm Hg is recommend for most adults 65 years and older in the 2017 American College of Cardiology/American Heart Association BP guideline,^29^ which contrasts with the American College of Physicians and American Academy of Family Physicians BP guideline where SBP less than 150 mm Hg is recommended in adults 60 years and older.^30^ These also differ from the 2018 European Society of Cardiology/European Society of Hypertension BP guideline, in which a BP of 130 to 139/70 to 79 mm Hg is considered.^31^ This is mainly due to the limited evidence and uncertain treatment effect from some trials^25,27,28^ in such population. Recently, the STEP trial^4^ showed that in patients aged 60 to 80 years with hypertension, targeting a reduction in SBP to 110 to 130 mm Hg resulted in a significantly lower incidence of MACE than a targeted reduction to 130 to 150 mm Hg. This was in line with the final report of SPRINT trial and its subgroup analysis report for patients 75 years and older.^32,33^ More recently, in a meta-analysis with more than 350 000 individual participants from 51 randomized clinical trials, pharmacological BP lowering to less than 120/70 mm Hg was effective into old age.^2^ This was consistent with our findings, which included more than 27 000 participants with a mean age of 70 years from 6 BP target trials.^4,24,25,26,27,28^

Even though the treatment thresholds differ between guidelines, clinicians are advised to determine BP targets based on a thorough review of comorbidities and patients’ life expectancy. A framework for individualizing prevention decisions incorporating TTB is being increasingly discussed. Analyzing and reporting this measurement would add more information about treatment effectiveness to clinicians’ evidence base.^7,8^ Previously, TTB was estimated through visually identifying the time point at which the curves separate.^8,34^ Clearly, this approach was subject to visual bias. Some other studies assessed the TTB by estimating the timing until the treatment effect reached statistical significance but heavily relied on an arbitrary *P* value.^35,36^ In the present study, we adopted the method proposed previously^9,11,12^ to calculate the time to reach the clinical meaningful ARR. Based on our analysis among populations with a mean age of 70 years, we found that it took 9.1 months on average to prevent 1 MACE in 500 people, suggesting that for most patients with a life expectancy of less than 1 year, the harms of intensive BP control may outweigh its benefits. Likewise, it took 34.4 months to prevent 1 MACE from intensive BP control for 100 patients, suggesting that for most patients with a life expectancy greater than 3 years, the benefits may likely outweigh the harms. Of note, a longer TTB was observed for trials with target SBP less than 120 mm Hg, which may reflect a higher cardiovascular disease (CVD) risk in the early period or immediate harms such as injurious falls, kidney disease, and other complications of hypotension following more aggressive BP treatment in comparison with target SBP less than 130 mm Hg, even though the treatment benefit was found at the end of the study.

The summary TTB results of the present study provided a global estimate for prevention with intensive BP control; individual patients may be best served by focusing on TTB results from studies with similar intensive BP control interventions or patient characteristics. The degree to which an individual patient will benefit from intensive BP control will likely depend on their risk profile and potential harm. The BP guidelines^29,30,31,37^ recommend that clinical judgment, patient preference, and a team-based approach to assess risk–benefit is reasonable for decisions regarding intensity of BP lowering and choice of antihypertensive drugs for older adults. The TTB as part of this information may help clinicians by providing a framework for therapeutic decisions to prioritize and individualize therapies to reduce CVD risk expeditiously. Meanwhile, it may also help patients with hypertension to recognize the potential for rapid benefit from the prescribed therapy.

### Limitations

First, although results from the SPRINT trial confirmed the benefit from intensive BP treatment among patients older than 75 years and did not show evidence of an interaction between treatment and age group,^32,33^ it was still unclear whether this benefit could be replicated in patients older than 80 years because this specific age group has been traditionally excluded or underrepresented in clinical trials. Thus, uncertainties regarding the TTB estimates may exist among this specific population. Second, although we performed several sensitivity analyses among these included trials, such as the exclusions of ACCORD BP trial^25^ with a diabetic population only or trials with kidney outcomes included in the definition of MACE, we may still not extensively assess the association with TTB estimates from the heterogeneities across trials. Third, the algorithm could not provide us with additional patient-level covariates for further detailed analyses by different characteristics (eg, sex, race and ethnicity, baseline CVD risk) even though it enabled us to closely approximate the original patient-level survival data within each individual study. Therefore, we were not sure whether patients at greater CVD risk might have a shorter TTB. Fourth, the concept of intensive BP interventions as well as its health complications among patients with hypertension has evolved tremendously in recent years. It is important to recognize the limitations of different comparators across different trials, which may affect our estimations. Finally, harms, such as injurious fall, hypotension, syncope, electrolyte abnormalities, and acute kidney injury or failure, have been reported to be associated with intensive BP treatment.^38,39^ A study from the SPRINT trial found that serious adverse events from intensive BP treatment (<120 mm Hg) were seen 9 months earlier than the decrease in MACE.^34^ However, the present study could not access these safety data to detail the time to harm. Therefore, further research is needed in this area.

## Conclusions

In this analysis, we found that treating 100 older patients (≥60 years) with hypertension for approximately 3 years would prevent 1 MACE. These findings suggests that intensive SBP control may be most appropriate for older adults with a life expectancy of greater than 3 years. For those with a life expectancy of less than 1 year, the harms of intensive BP control may outweigh the benefits. These results reinforce the importance of individualizing intensive BP control decisions by incorporating each patient’s values and preferences.

## Notes

Supplement. **eMethods.** Detailed description for TTB using frequentist method **eTable 1.** Reason for those excluded clinical trials (N=61) **eTable 2.** Component of MACE for each included trial **eTable 3.** Summarization of antihypertensive treatment strategies in each included trial **eTable 4.** Results of quality of reporting assessment using risk of bias using the Cochrane tool for assessment of risk of bias **eTable 5.** Time to benefit (months) for individual trial at specific thresholds of absolute risk reduction **eFigure 1.** Reconstruct KM curve and original KM curve (Cardio-Sis study) **eFigure 2.** Reconstruct KM curve and original KM curve (JATOS study) **eFigure 3.** Reconstruct KM curve and original KM curve (VALISH study) **eFigure 4.** Reconstruct KM curve and original KM curve (STEP study) **eFigure 5.** Reconstruct KM curve and original KM curve (SPRINT study) **eFigure 6.** Reconstruct KM curve and original KM curve (ACCORD study)
