# Supplementary material for: Time to Clinical Benefit of Intensive Blood Pressure Lowering in Patients 60 Years and Older With Hypertension: A Secondary Analysis of Randomized Clinical Trials
Source: JAMA Intern Med. 2022 May 9;182(6):660–7. doi: 10.1001/jamainternmed.2022.1657 (PMC9086939; doi:10.1001/jamainternmed.2022.1657)
Supplement: Supplement. — eMethods. Detailed description for TTB using frequentist method eTable 1. Reason for those excluded clinical trials (N=61) eTable 2. Component of MACE for each included trial eTable 3. Summarization of antihypertensive treatment strategies in each included trial eTable 4. Results of quality of reporting assessment using risk of bias using the Cochrane tool for assessment of risk of bias eTable 5. Time to benefit (months) for individual trial at specific thresholds of absolute risk reduction eFigure 1. Reconstruct KM curve and original KM curve (Cardio-Sis study) eFigure 2. Reconstruct KM curve and original KM curve (JATOS study) eFigure 3. Reconstruct KM curve and original KM curve (VALISH study) eFigure 4. Reconstruct KM curve and original KM curve (STEP study) eFigure 5. Reconstruct KM curve and original KM curve (SPRINT study) eFigure 6. Reconstruct KM curve and original KM curve (ACCORD study) [file jamainternmed-e221657-s001.pdf]

Supplemental Online Content

Chen T, Shao F, Chen K, et al. Time to clinical benefit of intensive blood pressure lowering in patients 60 years and older with hypertension: a secondary analysis of randomized clinical trials. *JAMA Intern Med*. Published online May 9, 2022. doi:10.1001/jamainternmed.2022.1657

eMethods. Detailed description for TTB using frequentist method

eTable 1. Reason for those excluded clinical trials(N=61)

eTable 2. Component of MACE for each included trial

eTable 3. Summarization of antihypertensive treatment strategies in each included trial

eTable 4. Results of quality of reporting assessment using risk of bias using the Cochrane tool for assessment of risk of bias

eTable 5. Time to benefit (months) for individual trial at specific thresholds of absolute risk reduction

eFigure 1. Reconstruct KM curve and original KM curve (Cardio-Sis study)

eFigure 2. Reconstruct KM curve and original KM curve (JATOS study)

eFigure 3. Reconstruct KM curve and original KM curve (VALISH study)

eFigure 4. Reconstruct KM curve and original KM curve (STEP study)

eFigure 5. Reconstruct KM curve and original KM curve (SPRINT study)

eFigure 6. Reconstruct KM curve and original KM curve (ACCORD study)

This supplemental material has been provided by the authors to give readers additional information about their work.

eMethods. Detailed description for TTB using frequentist method

Unlike the method implemented by Yourman etal[1], we calculated TTB and its confidence interval using conventional frequentist method with Monte Carlo simulations instead of the Bayesian approach. We fitted Weibull survival curves using the individual data for the control and intervention groups for each study. The R function "survreg" in the "survival" package was used to estimate the Weibull regression models with argument "strata()" for groups to make both the scale and shape parameters of Weibull distributions to vary for groups. TTB was obtained by numerically solve the equation to make the difference of the Weibull survival curves of groups estimated by the regression model to be the specific absolute risk reduction (ARR) threshold (i.e., 0.002, 0.005, and 0.010). The confidence interval of TTB was calculated based on the simulated sampling distribution of TTB by Monte Carlo method as follows. After the Weibull regression model was estimated, we simulated the Weibull distribution parameters by the estimated asymptotic sampling distribution of them, which was a multivariate normal distribution with the mean vector of parameter estimates and estimated variance covariance matrix. Simulated TTBs were calculated in the same manner described before with simulated Weibull parameters and were treated as the empirical sampling distribution of TTB. Afterwards, the confidence interval of TTB is calculated based on their quantile values. We found the empirical distribution of the logarithm of TTB is single-peaked and approximately symmetric. Therefore, the pivot type bootstrap confidence interval formula[2-4] was also implemented to make TTB's confidence interval more reliable.

In some studies, the calculated TTBs were too large to be practical. Thus, we considered to estimate an overall Weibull regression models for all studies with the study variable as a factor and the "strata()" argument. The overall Weibull survival curves estimated for different groups were based on the weighted averages of the estimated Weibull distribution parameters for each study, normalized by the sample size of each study as weights. The overall TTB and its confidence interval was calculated with the same method as before.

1. Yourman LC, Cenzer IS, Boscardin WJ, et al. Evaluation of Time to Benefit of Statins for the Primary Prevention of Cardiovascular Events in Adults Aged 50 to 75 Years: A Meta-analysis. *JAMA Intern Med.* 2021;181(2):179–185. doi:10.1001/jamainternmed.2020.6084
2. Efron B, Tibshirani RJ. An introduction to the Bootstrap. New York: Chapman & Hall; 1993.
3. Davision AC, Hinkley DV. Bootstrap methods and their application. Cambridge University Press; 1997.

© 2022 Chen T et al. *JAMA Intern Med.*

4. Carpenter J, Bithell J. Bootstrap confidence intervals: when, which, what? A practical guide for medical statisticians. *Stat Med*. 2000;19(9):1141-1164.

© 2022 Chen T et al. *JAMA Intern Med.*

eTable 1. Reason for those excluded clinical trials(N=61)

Number	Title	Reasons of exclusion
1	A support programme for secondary prevention in patients with transient ischaemic attack and minor stroke (INSPiRE-TMS): an open-label, randomised controlled trial.	Not treat-to-target design
2	Allisartan Isoproxil Improves Endothelial Function and Vascular Damage in Patients with Essential Hypertension: A Single-Center, Open-Label, Randomized Controlled Trial.	Not treat-to-target design
3	Promoting Physical Activity in Older Adults With Knee Osteoarthritis and Hypertension: A Randomized Controlled Trial.	Not treat-to-target design
4	Long-Term Effects of an Intensive Prevention Program After Acute Myocardial Infarction.	Not treat-to-target design
5	Systolic Blood Pressure in Heart Failure With Preserved Ejection Fraction Treated With Sacubitril/Valsartan.	Not treat-to-target design
6	ANICHKOV study: the effect of combined hypotensive and lipid-lowering therapy on cardiovascular complications in patients of high and very high risk.	Not treat-to-target design
7	A comparison of outcomes with angiotensin-converting--enzyme inhibitors and diuretics for hypertension in the elderly.	Not treat-to-target design
8	Results of the pilot study for the Hypertension in the Very Elderly Trial.	Not treat-to-target design
9	High-intensity, whole-body exercise improves blood pressure control in individuals with spinal cord injury: A prospective randomized controlled trial.	Not treat-to-target design
10	Static Stretch Performed After Strength Training Session Induces Hypotensive Response in Trained Men.	Not treat-to-target design
11	Assessment of Losartan 50 mg on Survival of Post-Dialysis Euvolemic Hypertensive Patients: Findings from HELD Trial.	Not treat-to-target design
12	Cardiac output and cerebral blood flow during carotid surgery in regional versus general anesthesia: A prospective randomized controlled study.	Not treat-to-target design
13	Effect of a Coordinated Community and Chronic Care Model Team Intervention vs Usual Care on Systolic Blood Pressure in Patients With Stroke or Transient Ischemic Attack: The SUCCEED Randomized Clinical Trial.	Not treat-to-target design
14	Reducing Hypertension in a Poststroke Black and Hispanic Home Care Population: Results of a Pragmatic Randomized Controlled Trial.	Not treat-to-target design
15	A randomized control trial comparing prophylactic dexmedetomidine versus clonidine on rates and duration of delirium in older adult patients undergoing coronary artery bypass grafting.	Not treat-to-target design
16	An electronic decision support-based complex intervention to improve management of cardiovascular risk in primary health care: a cluster randomised trial (INTEGRATE).	Not treat-to-target design
17	Effects of an Electronic Software "Prompt" With Health Care Professional Training on Cardiovascular and Renal Complications in a Multiethnic Population With Type 2 Diabetes and Microalbuminuria (the GP-Prompt Study).	Not treat-to-target design
18	Effects of allisartan isoproxil on blood pressure and target organ injury in patients with mild to moderate essential hypertension.	Not treat-to-target design

© 2022 Chen T et al. *JAMA Intern Med.*

19	Prevention of stroke by antihypertensive drug treatment in older persons with isolated systolic hypertension. Final results of the Systolic Hypertension in the Elderly Program (SHEP). SHEP Cooperative Research Group.	Not treat-to-target design
20	Medical Research Council trial of treatment of hypertension in older adults: principal results.	Not treat-to-target design
21	Randomized double-blind comparison of a calcium antagonist and a diuretic in elderly hypertensives.	Not treat-to-target design
22	Randomised trial of a perindopril-based blood-pressure-lowering regimen among 6,105 individuals with previous stroke or transient ischaemic attack.	Not treat-to-target design
23	Major outcomes in high-risk hypertensive patients randomized to angiotensin-converting enzyme inhibitor or calcium channel blocker vs diuretic: The Antihypertensive and Lipid-Lowering Treatment to Prevent Heart Attack Trial (ALLHAT).	Not treat-to-target design
24	Mortality and morbidity results from the European Working Party on High Blood Pressure in the Elderly trial.	Not treat-to-target design
25	Treatment of hypertension in patients 80 years of age or older.	Not treat-to-target design
26	Effects of losartan on renal and cardiovascular outcomes in patients with type 2 diabetes and nephropathy.	Not treat-to-target design
27	A randomised double-blind pilot trial comparing a mean arterial pressure target of 65 mm Hg versus 72 mm Hg after out-of-hospital cardiac arrest.	Not treat-to-target design
28	Positive Health Beliefs and Blood Pressure Reduction in the DESERVE Study.	Not treat-to-target design
29	Mortality and morbidity during and after the Antihypertensive and Lipid-Lowering Treatment to Prevent Heart Attack Trial.	Not treat-to-target design
30	Morbidity and mortality in the Swedish Trial in Old Patients with Hypertension (STOP-Hypertension).	Not treat-to-target design
31	Lowering blood pressure reduces renal events in type 2 diabetes.	Not treat-to-target design
32	Antihypertensive efficacy and side effects of three beta-blockers and a diuretic in elderly hypertensives: a report from the STOP-Hypertension study.	Not treat-to-target design
33	Shanghai trial of nifedipine in the elderly (STONE).	Not treat-to-target design
34	Randomised trial of old and new antihypertensive drugs in elderly patients: cardiovascular mortality and morbidity the Swedish Trial in Old Patients with Hypertension-2 study.	Not treat-to-target design
35	Randomised double-blind comparison of placebo and active drugs for effects on risks associated with blood pressure variability in the Systolic Hypertension in Europe trial.	Not treat-to-target design
36	Comparison of antihypertensive treatments in preventing cardiovascular events in elderly diabetic patients: results from the Swedish Trial in Old Patients with Hypertension-2.	Not treat-to-target design
37	The Study on Cognition and Prognosis in the Elderly (SCOPE): principal results of a randomized double-blind intervention trial.	Not treat-to-target design
38	Comparison of active treatment and placebo in older Chinese patients with isolated systolic hypertension. Systolic Hypertension in China (Syst-China) Collaborative Group.	Not treat-to-target design
39	The Felodipine Event Reduction (FEVER) Study: a randomized long-term placebo-controlled trial in Chinese hypertensive patients.	Not treat-to-target design

© 2022 Chen T et al. *JAMA Intern Med.*

40	Treatment of isolated systolic hypertension: The SHELL study results.	Not treat-to-target design
41	Renin and Survival in Patients Given Angiotensin II for Catecholamine-Resistant Vasodilatory Shock. A Clinical Trial.	Not treat-to-target design
42	Angiotensin II receptor blocker-based therapy in Japanese elderly, high-risk, hypertensive patients.	Not treat-to-target design
43	Association of Sustained Blood Pressure Control with Multimorbidity Progression Among Older Adults.	Not treat-to-target design
44	Effects of a fixed combination of perindopril and indapamide on macrovascular and microvascular outcomes in patients with type 2 diabetes mellitus (the ADVANCE trial): a randomised controlled trial.	Not treat-to-target design
45	Morbidity and mortality in the Systolic Hypertension in the Elderly Program (SHEP) pilot study.	Not treat-to-target design
46	Randomised double-blind comparison of placebo and active treatment for older patients with isolated systolic hypertension.	Not treat-to-target design
47	Chinese trial on isolated systolic hypertension in the elderly.	Not treat-to-target design
48	Effect of amlodipine on morbidity and mortality in severe chronic heart failure.	Not treat-to-target design
49	The perindopril in elderly people with chronic heart failure (PEP-CHF) study.	Not treat-to-target design
50	Restoration of nocturnal blood pressure dip and reduction of nocturnal blood pressure with evening anti-hypertensive medication administration in pediatric kidney transplant recipients: A pilot randomized clinical trial.	Not treat-to-target design
51	Timolol-induced reduction in mortality and reinfarction in patients surviving acute myocardial infarction.	Not treat-to-target design
52	Comparative effects of intensive-blood pressure versus standard-blood pressure-lowering treatment in patients with severe ischemic stroke in the ENCHANTED trial	Participants are not the elderly
53	Treating Home Versus Predialysis Blood Pressure Among In-Center Hemodialysis Patients: A Pilot Randomized Trial.	Participants are not the elderly
54	Blood-pressure targets in patients with recent lacunar stroke: the SPS3 randomised trial.	Participants are not the elderly
55	Randomized trial of an increased dose of calcium channel blocker or angiotensin II type 1 receptor blocker as an add-on intensive depressor therapy in type 2 diabetes mellitus patients with uncontrolled essential hypertension: the ACADEMIE Study.	Participants are not the elderly
56	Effects of Blood Pressure Lowering on Clinical Outcomes According to Baseline Blood Pressure and Cardiovascular Risk in Patients With Type 2 Diabetes Mellitus.	Participants are not the elderly
57	Tight blood pressure control and risk of macrovascular and microvascular complications in type 2 diabetes: UKPDS 38. UK Prospective Diabetes Study Group.	Participants are not the elderly
58	The BBB Study: the effect of intensified antihypertensive treatment on the level of blood pressure, side-effects, morbidity and mortality in "well-treated" hypertensive patients. Behandla Blodtryck Bättre.	Participants are not the elderly
59	Initial treatment with a single pill containing quadruple combination of quarter doses of blood pressure medicines versus standard dose monotherapy in patients with hypertension (QUARTET): a phase 3, randomised, double-blind, active-controlled trial.	Participants are not the elderly
60	Effects of intensive blood-pressure lowering and low-dose aspirin in patients with hypertension: principal results of the Hypertension Optimal Treatment (HOT) randomised trial.	No KM curves showed

© 2022 Chen T et al. *JAMA Intern Med.*

61	Effects of Intensive Antihypertensive Treatment on Chinese Hypertensive Patients Older Than 70 Years.	No risk table showed in the KM curves

© 2022 Chen T et al. *JAMA Intern Med.*

eTable 2. Component of MACE for each included trial

Outcomes	Included studies					
	SPRINT	ACCORD	VALISH	Cardio-Sis	JATOS	STEP
**Heart**						
Myocardial infarction	√	√	√	√	√	×
Acute coronary syndromes	√	×	×	×	×	√
Angina pectoris	×	×	×	√	√	×
Coronary revascularization	×	×	×	√	×	√
Heart failure	√	×	×	√	√	√
Unplanned hospitalization for cardiovascular disease	×	×	√	×	×	×
Dissecting aneurysms of the aorta	×	×	×	×	√	×
Aortic dissection	×	×	×	√	×	×
Atrial fibrillation	×	×	×	√	×	√
Occlusive arterial disease	×	×	×	√	√	×
**Brain**						
Stroke	√	√	√	√	×	√
Cerebral infarction	×	×	×	×	√	×
Transient ischaemic attack	×	×	×	√	√	×
Cerebral hemorrhage	×	×	×	×	√	×
Subarachnoid hemorrhage	×	×	×	×	√	×
**Kidney**						
Renal failure	×	×	×	√	√	×
Renal dysfunction	×	×	√	×	×	×
**Death**						
All-cause mortality	×	×	×	√	×	×
Sudden death	×	×	√	×	√	×
Death from cardiovascular causes	√	√	√	×	√	√
Death because of heart failure	×	×	√	×	×	×

MACE: Major adverse cardiovascular events;

MACE definition:
SPRINT: Myocardial infarction, other acute coronary syndromes, stroke, heart failure, or death from cardiovascular causes;

ACCORD: nonfatal myocardial infarction, nonfatal stroke, or cardiovascular death;

VALISH: cardiovascular events (sudden death, fatal or nonfatal stroke, fatal or nonfatal myocardial infarction, death because of heart failure, other cardiovascular death, unplanned hospitalization for cardiovascular disease), and renal dysfunction (doubling of serum creatinine to a level >2.0 mg per 100 mL or introduction of dialysis);

Cardio-Sis: All-cause mortality, fatal or non-fatal myocardial infarction, fatal or non-fatal stroke, transient ischaemic attack, congestive heart failure of New York Heart Association stages III or IV requiring admission to hospital, angina pectoris with objective evidence of myocardial ischaemia, new-onset atrial fibrillation,

© 2022 Chen T et al. *JAMA Intern Med.*

coronary revascularisation, aortic dissection, occlusive peripheral arterial disease, and renal failure requiring dialysis.

JATOS: Combined incidence of cerebrovascular disease (cerebral hemorrhage, cerebral infarction, transient ischemic attack, and subarachnoid hemorrhage), cardiac and vascular disease (myocardial infarction, angina pectoris requiring hospitalization, heart failure, sudden death, dissecting aneurysms of the aorta, and occlusive arterial disease), and renal failure (acute or chronic renal failure; doubling of the serum creatinine concentration to a value of 1.5 mg/dL or higher);

STEP: Stroke, acute coronary syndrome, acute decompensated heart failure, coronary revascularization, atrial fibrillation, or death from cardiovascular causes.

© 2022 Chen T et al. *JAMA Intern Med.*

eTable 3. Summarization of antihypertensive treatment strategies in each included trial

Included studies	Antihypertensive Treatment Strategies
VALISH	Valsartan, 40 to 80 mg once daily, was administrated as the first-step therapy. If the target BP in each group was not achieved within 1 to 2 months, the dose of valsartan was increased ≤ 160 mg, and/or other antihypertensive agents except other angiotensin II type 1 receptor blockers were added, for example, low-dose diuretics, Ca antagonists, and so on to maintain the target BP.
JATO	Untreated subjects initially received efonidipine at a daily dose of 20 to 40 mg (once daily). In subjects who were already receiving antihypertensive medications, a similar dose of efonidipine was added or substituted for one of the drugs being received before study entry without a washout period. The daily dose of efonidipine could be increased to 60 mg (once or twice daily) and antihypertensive drugs other than calcium antagonists were added, if needed.
Cardio-Sis	Antihypertensive drug treatment included various combinations of previous drugs (background therapy) plus drugs made available for the purpose of the study. We dispensed furosemide (25 mg per day), ramipril (5 mg or 10 mg per day), telmisartan (80 mg per day), amlodipine (5 mg or 10 mg per day), bisoprolol (5 mg per day), and transdermal clonidine (2.5 mg or 5.0 mg per day). Ramipril and telmisartan were also available in fixed combinations with hydrochlorothiazide (12.5 mg or 25.0 mg per day for ramipril, and 12.5 mg per day for telmisartan).
SPRINT	All major classes of antihypertensive agents were included in the formulary and were provided at no cost to the participants. SPRINT investigators could also prescribe other antihypertensive medications (not provided by the study). The protocol encouraged, but did not mandate, the use of drug classes with the strongest evidence for reduction in cardiovascular outcomes, including thiazide-type diuretics (encouraged as the first-line agent), loop diuretics (for participants with advanced chronic kidney disease), and beta-adrenergic blockers (for those with coronary artery disease).Chlorthalidone was encouraged as the primary thiazide-type diuretic, and amlodipine as the preferred calcium-channel blocker.
ACCORD	All major classes of antihypertensive drugs and many combination medications were provided by the study. All antihypertensive regimens were to include a drug class that had demonstrated reduced cardiovascular events in participants with diabetes: diuretic, beta-blocker, calcium channel blocker (CCB), angiotensin converting-enzyme (ACE) inhibitor, or angiotensin receptor blocker (ARB).For intensive participants, a combination of a diuretic and either an ACE inhibitor or a beta-blocker was recommended as initial therapy.
STEP	After randomization, all patients were scheduled for follow-up visits at 1, 2, and 3 months and every 3 months thereafter until 48 months. The patients were provided with antihypertensive drugs, including olmesartan (an angiotensin-receptor blocker), amlodipine (a calcium-channel blocker), and hydrochlorothiazide (a diuretic).

© 2022 Chen T et al. *JAMA Intern Med.*

eTable 4. Results of quality of reporting assessment using risk of bias using the Cochrane tool for assessment of risk of bias

	**JATOS**	**SPRINT**	**VALISH**	**Cardio-Sis**	**ACCORD**	**STEP**
Random sequence generation	Low	Low	Low	Low	Low	Low
Allocation concealment	Low	Low	Low	Low	Low	Low
Blinding of participants and researchers	Unclear	Unclear	Unclear	Unclear	Unclear	Unclear
Blinding of outcome assessment	Low	Low	Low	Low	Low	Low
Incomplete outcome data	Low	Low	Low	Low	Low	Low
Selective reporting	Low	Low	Low	Low	Low	Low
Other bias	Low	Low	Low	Low	Low	Low

© 2022 Chen T et al. *JAMA Intern Med.*

eTable 5. Time to benefit (months) for individual trial at specific thresholds of absolute risk reduction

	SPRINT	ACCORD	CARDIO-SIS	JATOS	VALISH	STEP
Threshold						
0.002	17.1(1.1, 19.5)	23.6(1.8, 43.6)	0.2(0.1, 2.0)	26.4(0.7, 81.4)	3.5(0.8, 63.4)	3.4(1.4, 44.7)
0.005	23.0(5.3, 38.7)	35.8(5.1, 86.2)	0.7(0.2, 9.8)	48.5(2.8, 219.5)	131.9(2.9, 152.7)	11.3(0.5, 27.3)
0.01	31.3(16.6, 55.4)	52.6(20.2, 240.8)	1.9(0.6, 47.0)	83.5(15.1, 895.0)	165.6(8.9, 294.5)	30.6(12.0, 712.7)

© 2022 Chen T et al. *JAMA Intern Med.*

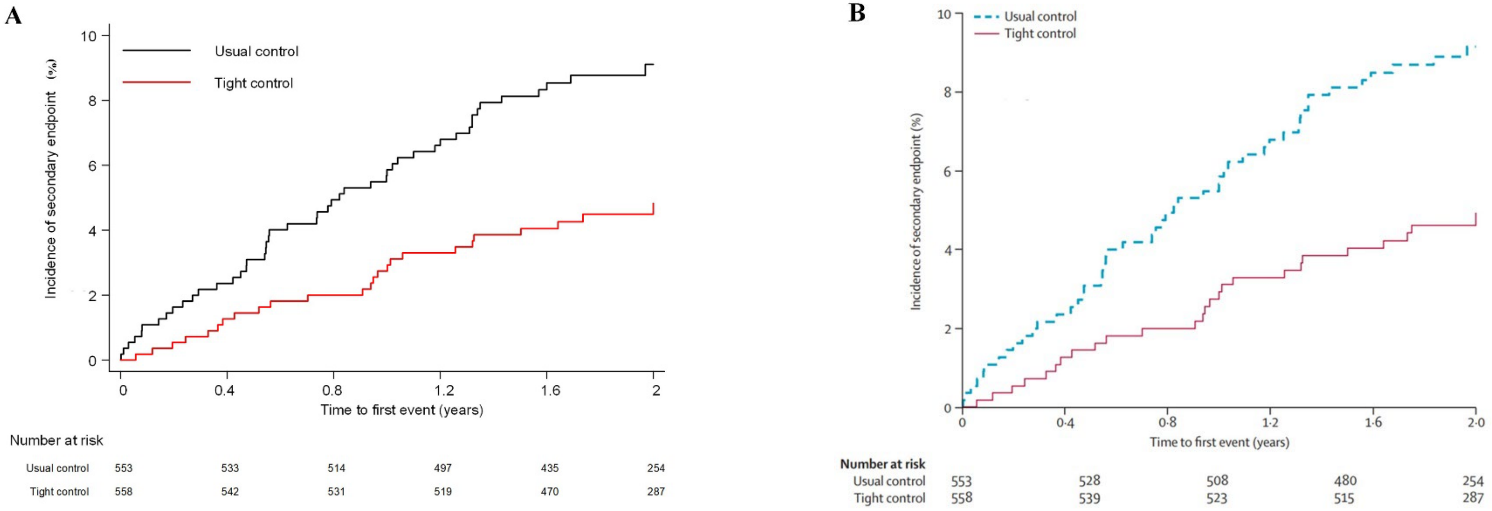

eFigure 1. Reconstruct KM curve and original KM curve (Cardio-Sis study)

(A) Reconstruct KM curve, (B) Original KM curve

© 2022 Chen T et al. *JAMA Intern Med.*

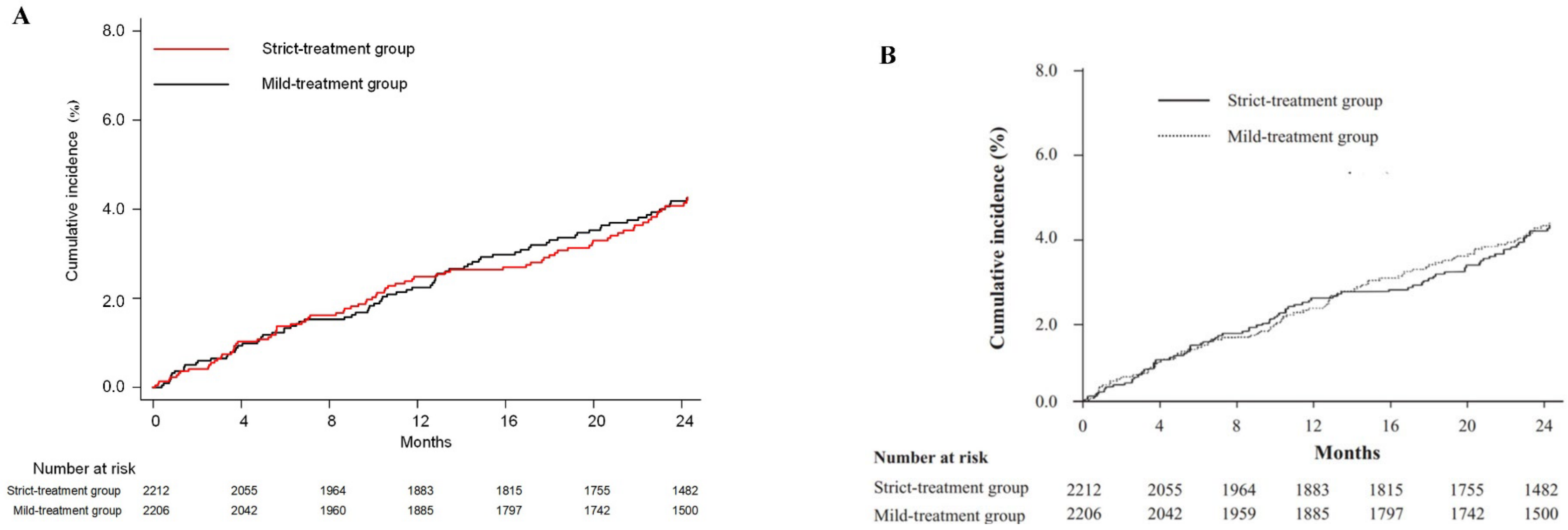

eFigure 2. Reconstruct KM curve and original KM curve (JATOS study)

(A) Reconstruct KM curve, (B) Original KM curve

© 2022 Chen T et al. *JAMA Intern Med.*

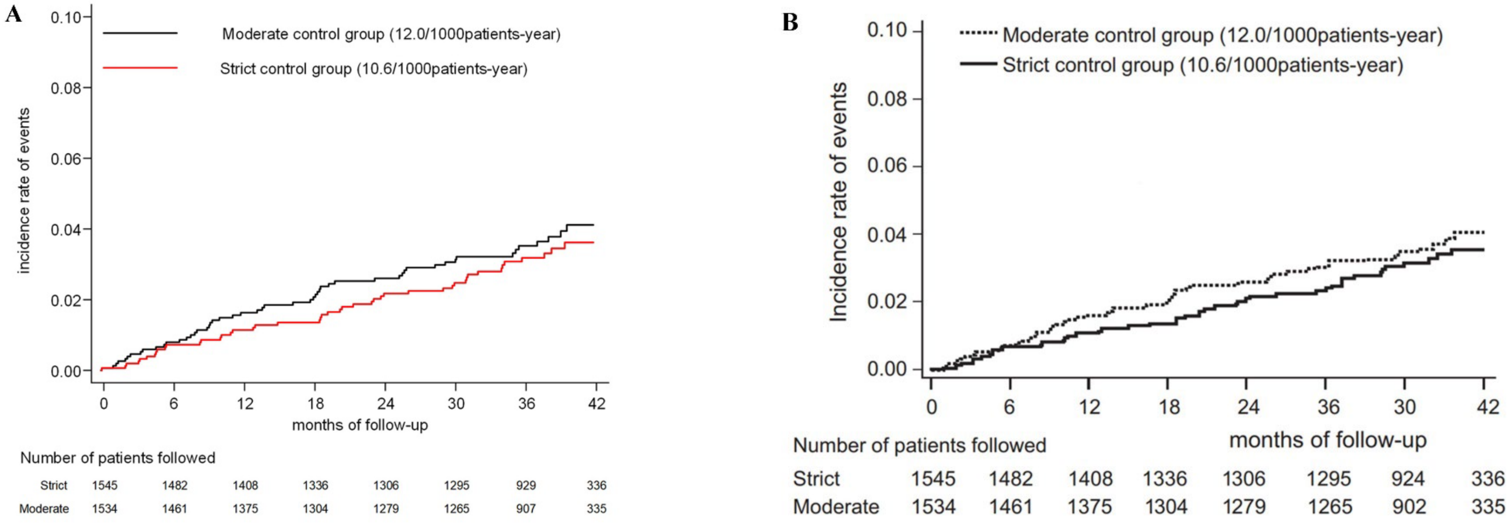

eFigure 3. Reconstruct KM curve and original KM curve (VALISH study)

(A) Reconstruct KM curve, (B) Original KM curve

© 2022 Chen T et al. *JAMA Intern Med.*

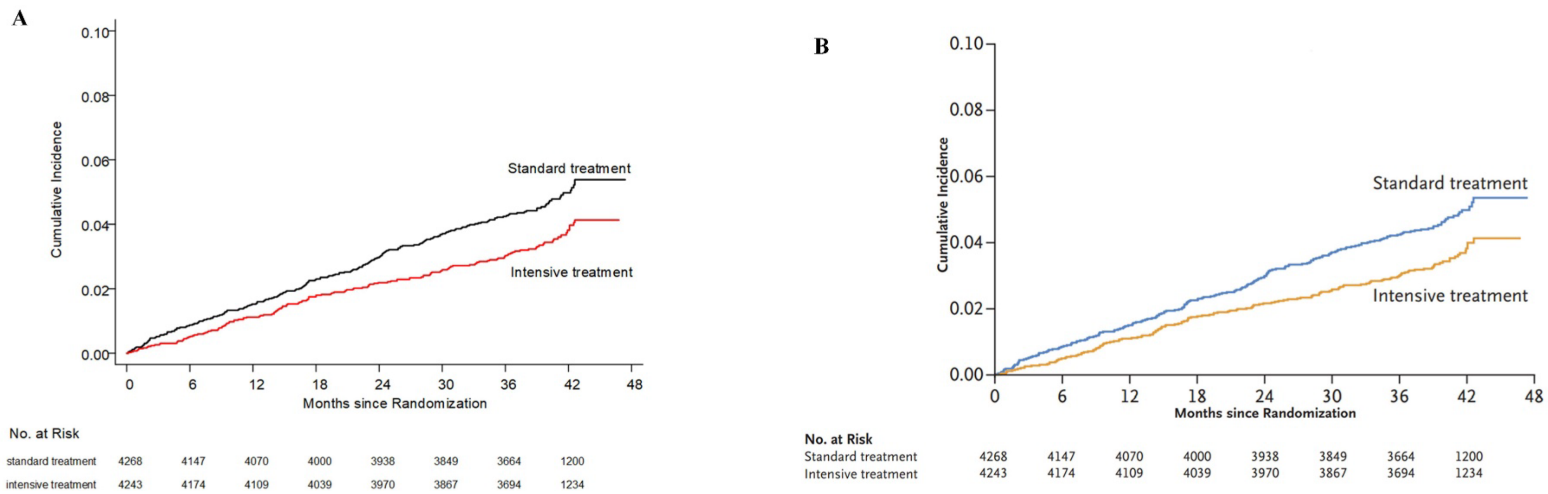

eFigure 4. Reconstruct KM curve and original KM curve (STEP study)

(A) Reconstruct KM curve, (B) Original KM curve

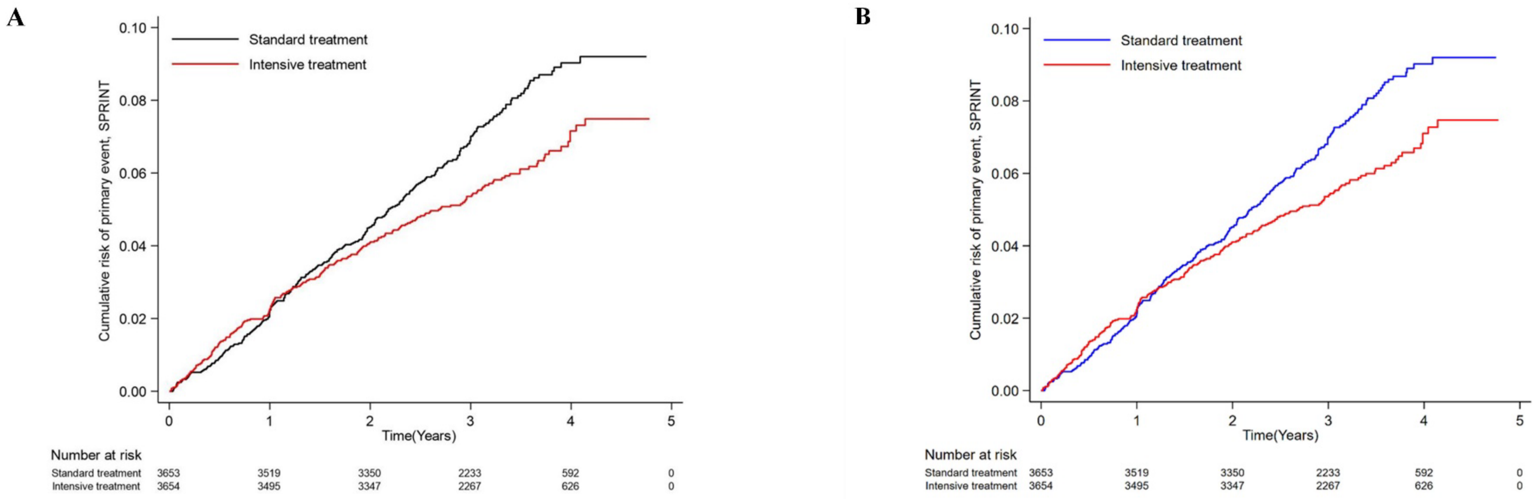

eFigure 5. Reconstruct KM curve and original KM curve (SPRINT study)

(A) Reconstruct KM curve, (B) Original KM curve

© 2022 Chen T et al. *JAMA Intern Med.*

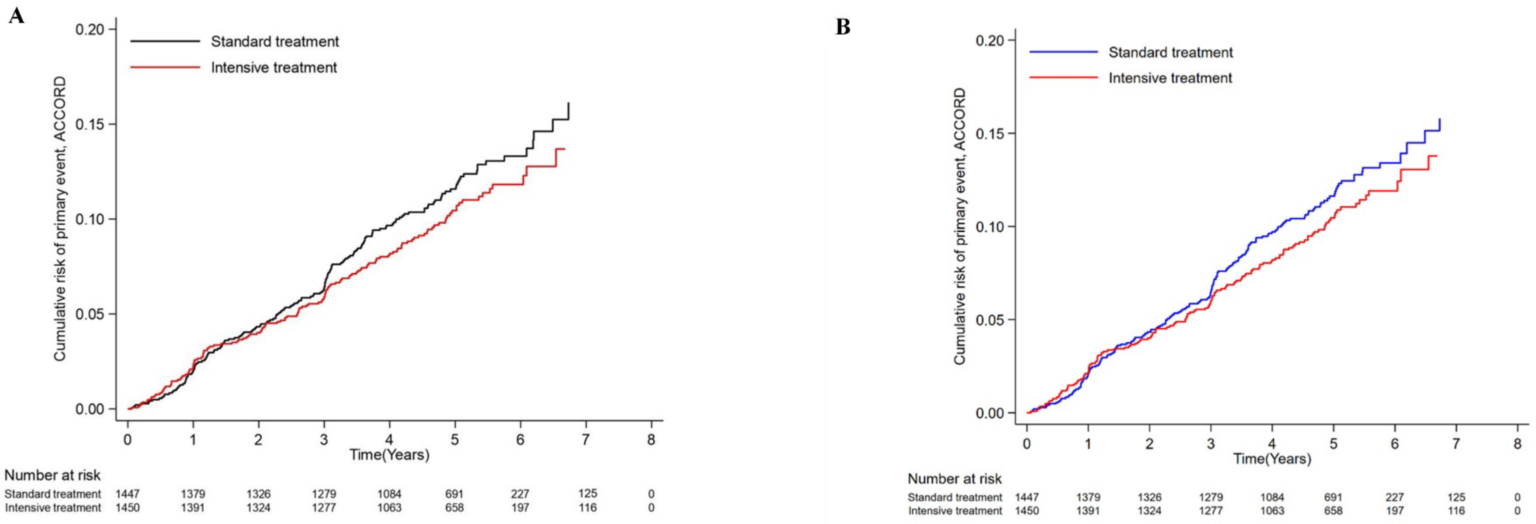

eFigure 6. Reconstruct KM curve and original KM curve (ACCORD study)

(A) Reconstruct KM curve, (B) Original KM curve

© 2022 Chen T et al. *JAMA Intern Med.*
